# A research data management (RDM) community for ELIXIR

**DOI:** 10.12688/f1000research.146301.2

**Published:** 2024-09-30

**Authors:** Flora D'Anna, Niclas Jareborg, Mijke Jetten, Minna Ahokas, Pinar Alper, Robert Andrews, Korbinian Bösl, Teresa D’Altri, Daniel Faria, Nazeefa Fatima, Siiri Fuchs, Clare Garrard, Wei Gu, Katharina F. Heil, Yvonne Kallberg, Flavio Licciulli, Nils-Christian Lübke, Ana M. P. Melo, Ivan Mičetić, Jorge Oliveira, Anastasis Oulas, Patricia M. Palagi, Krzysztof Poterlowicz, Xenia Perez-Sitja, Patrick Ruch, Susanna-Assunta Sansone, Helena Schnitzer, Celia van Gelder, Thanasis Vergoulis, Daniel Wibberg, Ulrike Wittig, Brane Leskošek, Jiri Vondrasek, Munazah Andrabi

**Affiliations:** 1 VIB Data Core, VIB Technologies, Ghent, 75 9052, Belgium; 2 National Bioinformatics Infrastructure (NBIS), Department of Cell and Molecular Biolog,, Uppsala University, Uppsala, Sweden; 3 Stichting Health-RI, Utrecht, Jaarbeursplein 6, AL, 3521, Netherlands Antilles; 4 CSC – IT CENTER FOR SCIENCE LTD, Espoo, FI-02101, Finland; 5 Luxembourg National Data Service, Luxembourg, L- 4362, Luxembourg; 6 School of Medicine, Cardiff University, Cardiff, Wales, CF14 4YS, UK; 7 Department of Informatics, University of Bergen, Bergen, Norway; 8 Centre for Genomic Regulation (CRG), The Barcelona Institute of Science and Technology, Barcelona, 08003, Spain; 9 INESC-ID, IST, University of Lisbon, Lisbon, Portugal; 10 Centre for Bioinformatics, University of Oslo, Oslo, Norway; 11 ELIXIR Hub, Wellcome Trust Genome Campus, Hinxton, Cambridge, CB10 1SD, UK; 12 NBIS, SciLifeLab, Stockholm University, Stockholm, Sweden; 13 Institute for Biomedical Technologies, Bari, 70126, Italy; 14 Forschungszentrum Jülich GmbH, Jülich, 52428, Germany; 15 Dept. of Biomedical Sciences, University of Padua, Padova, 35131, Italy; 16 BioData.pt - Portuguese Infrastructure of Biological Data, Oeiras, 2780-156, Portugal; 17 The Cyprus Institute of Neurology and Genetics, Nicosia, Cyprus; 18 SIB Swiss Institute of Bioinformatics, University of Lausanne, Lausanne, 1015, Switzerland; 19 Faculty of Life Sciences, CompData, University of Bradford, Bradford, England, BD7 1DP, UK; 20 Swiss Institute of Bioinformatics, University of Geneva, Geneva, 1206, Switzerland; 21 Information Sciences, HES-SO\HEG Genève, Geneva, Switzerland; 22 Oxford e-Research Centre, Department of Engineering Science, University of Oxford, Oxford, England, OX13QG, UK; 23 Athena Research Center, Athens, Greece; 24 Heidelberg Institute for Theoretical Studies, Heidelberg, 69118, Germany; 25 Institute for Biostatistics and Medical Informatics, Faculty of Medicine, University of Ljubljana, Ljubljana, Sl-1000, Slovenia; 26 Institute of Organic Chemistry and Biochemistry of the CAS, Prague, Czech Republic; 27 Computer Science, The University of Manchester, Manchester, England, M13 9PL, UK

**Keywords:** Data management, Data stewardship, Data management plans, FAIR principles, community standards, Data management training, Research data life cycle, Common best practices

## Abstract

Research data management (RDM) is central to the implementation of the FAIR (Findable Accessible, Interoperable, Reusable) and Open Science principles. Recognising the importance of RDM, ELIXIR Platforms and Nodes have invested in RDM and launched various projects and initiatives to ensure good data management practices for scientific excellence. These projects have resulted in a rich set of tools and resources highly valuable for FAIR data management. However, these resources remain scattered across projects and ELIXIR structures, making their dissemination and application challenging. Therefore, it becomes imminent to coordinate these efforts for sustainable and harmonised RDM practices with dedicated forums for RDM professionals to exchange knowledge and share resources.

The proposed ELIXIR RDM Community will bring together RDM experts to develop ELIXIR’s vision and coordinate its activities, taking advantage of the available assets. It aims to coordinate RDM best practices and illustrate how to use the existing ELIXIR RDM services. The Community will be built around three integral pillars, namely, a network of RDM professionals, RDM knowledge management and RDM training expertise and resources. It will also engage with external stakeholders to leverage benefits and provide a forum to RDM professionals for regular knowledge exchange, capacity building and development of harmonised RDM practices, keeping in line with the overall scope of the RDM Community.

In the short term, the Community aims to build upon the existing resources and ensure that the content of these remain up to date and fit for purpose. In the long run, the Community will aim to strengthen the skills and knowledge of its RDM professionals to support the emerging needs of the scientific community. The Community will also devise an effective strategy to engage with other ELIXIR structures and international stakeholders to influence and align with developments and solutions in the RDM field.

## Introduction

In the 2017 position paper on FAIR Data Management in the Life Sciences ( Blomberg, 2017), ELIXIR emphasised Findable, Accessible, Interoperable and Reusable (FAIR) ( Wilkinson *et al.*, 2016) data management as a crucial part of good scientific practice and research excellence, which requires professional skills and adequate resources. ELIXIR’s mission is to implement standards-based, FAIR data stewardship within European life science projects and to help its users to comply with FAIR and Open Science principles via the ELIXIR Nodes that support the FAIR data management needs of national research projects.

The ELIXIR position paper on FAIR Data Management in the life sciences also states that “ELIXIR Nodes are the national implementation of a harmonised FAIR Data Management programme for the life science” ( Blomberg, 2017), relying on the coordinated action of national Nodes to ensure harmonised data management practices. So far, initiatives to promote harmonisation of practices across Nodes have been conducted within the temporal limits of projects or within different ELIXIR structures (Nodes, Platforms, Communities, Focus Groups and the Staff Exchange Programme).

Since research funders started to require data management plans (DMPs) and the implementation of FAIR and Open Science principles, several Nodes have invested in data stewardship ( Carraro *et al.*, 2022). A high return on investment of data stewardship has been identified for research in general ( Mons, 2020), and managing data has been recognized as a matter of infrastructure, institutions, and economics, instead of simply an individual practice ( Borgman & Bourne, 2021). Nevertheless, there is no consensus on the understanding of what a data steward actually does, the context in which they operate, and the position of the data steward within institutions ( Jetten *et al.*, 2021). ELIXIR Nodes have been involved in national initiatives aimed at defining the role and the competences of a data steward. For instance, the National Programme Open Sciences (NPOS) and ELIXIR Netherlands developed a competency framework for data stewards, published in the EBI Competency Hub ( Scholtens *et al.*, 2019). Although this framework has been adopted by various ELIXIR Nodes, a long-lasting context to discuss, harmonise and update data steward job profiles and competences is still missing.

Members from ELIXIR Nodes have been involved in RDM related activities carried out via Staff Exchange Programmes, Focus Groups (e.g. EOSC, RDA Activities, FAIR Training), Platforms, Communities (e.g. Plant Science s) and projects. The ELIXIR Interoperability Platform, in particular, has also operated in the RDM space, focussing on the services and their use in interoperability stories. The Software Development Best Practices Working Group of the ELIXIR Tools Platform developed the Software Management Plan (SMP) template. The ELIXIR-CONVERGE project created a strong network of data stewards and domain and training experts. Knowledge resources for RDM best practices, such as RDMkit and the Data Stewardship Wizard (DSW) ( Pergl *et al.*, 2019), have been built, enriched and integrated with ELIXIR registries and offer an excellent body of knowledge to apply FAIR data management to life sciences research. Moreover, training resources on RDM have been developed and made findable via the ELIXIR Training Portal TeSS. The FAIRplus project connected ELIXIR with industry partners by developing key resources, including the FAIR Cookbook ( Rocca-Serra *et al.*, 2022) to implement FAIR principles and tools to evaluate FAIRness of datasets. While these initiatives have built the foundations of strategic ambitions in data management, there is currently no mechanism in ELIXIR to sustain and strengthen them beyond the stipulated duration of the projects.

In this white paper, we explain how the ELIXIR Research Data Management (RDM) Community is of benefit to the broader ELIXIR landscape, including its Nodes, Platforms, Communities and ultimately scientists (end-users), with a coherent approach to data management. The establishment of the RDM Community aligns tightly with the ELIXIR Scientific Programme 2024-2028, which adopts the outcomes of ELIXIR-CONVERGE, FAIRplus and other projects. The RDM Community further develops and strengthens the networks of data management experts from these two main projects, the training and capacity building efforts, and data management knowledge for the ELIXIR community and beyond. Essential to the ELIXIR RDM Community is the strengthening and consolidation of these and other relevant ELIXIR data management resources. These resources include tools, best practices and expertise within the ELIXIR Platforms and Communities, and the collaboratively developed ELIXIR training and capacity building resources aimed at the broader community for reuse and adoption. The ELIXIR RDM Community as a unified long-term framework for RDM professionals across Europe to come together and exchange knowledge is the key to the implementation of harmonised data management practices across Nodes. The RDM Community is meant to be inclusive to anyone that has a professional interest in RDM for the life sciences in academia and industry both in and outside of ELIXIR.

**Definition of terms.**

Although FAIR management often refers to *data*, the same principles apply to other research objects ( Jiménez *et al.*, 2017; Hong *et al.*, 2022; Garcia *et al.*, 2020), such as software and training materials. Thus, when referring to data, in this white paper, we also refer to other research objects. Since *data management* and *data stewardship* are newly evolving expertise areas, and there are no universally accepted definitions yet, in this white paper we will use both terms interchangeably. Our working definition is the one put forward by the Dutch Techcentre for Life Sciences (DTL/ELIXIR Netherlands): “Data stewardship is responsible planning and executing of all actions on digital data before, during and after a research project, with the aim of optimising the usability, reusability and reproducibility of the resulting data”.

## Landscape of RDM in ELIXIR

### RDM professionals

As a consequence of the implementation of DMPs, FAIR and open data policies at the European, national and institutional levels, a portfolio of RDM tools and services in each ELIXIR Node has become inevitable. In fact, based on a survey in March 2022 ( Carraro *et al.*, 2022), at least 17 Nodes offer services related to data management and stewardship. 75% of these Nodes predict that the RDM services they offer are likely to be sustainable over time, indicating a clear ambition to continue providing these services. The type of services offered vary greatly from guidelines and consultancy to IT infrastructures and training. RDM services are requested and used by scientific and technical staff (e.g. researchers, core facilities, data stewards, consortia for projects etc.) from universities, research institutions and industry.

**Figure 1.**
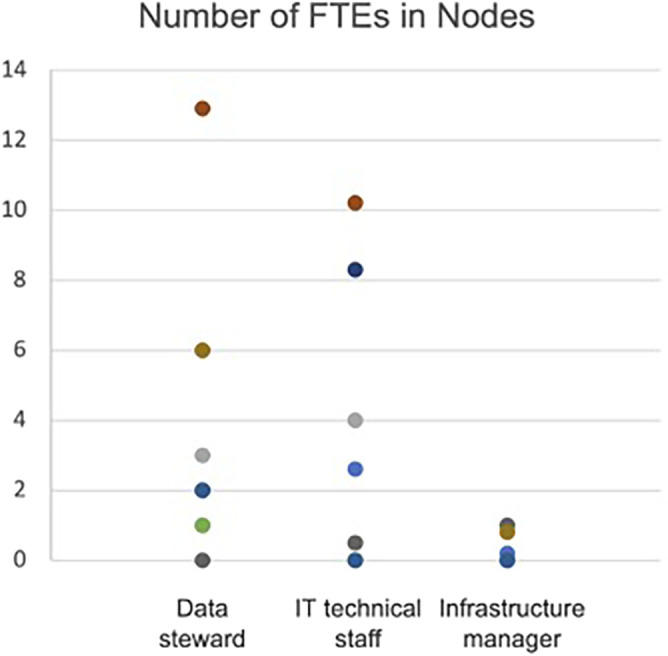
Number of full-time equivalents (FTEs) in Nodes providing RDM related services in 2021. Colours represent different ELIXIR Nodes. Data derived from the online surveys and interviews performed in the context of ELIXIR-CONVERGE ( Carraro *et al.*, 2022), investigating how services of data management and stewardship are delivered and financially supported in ELIXIR Nodes.

Based on the same survey, with answers from 11 Nodes, it seems that, on average, a Node itself has 2.9 FTE (full-time equivalents) as data stewards, 2.4 FTE as IT technical staff and 0.3 FTE as infrastructure managers ( Figure 1). Despite a considerable variation in the range of the number of FTEs among the Nodes (ranging from 1 to >5 FTE), this data illustrates the diversity of national environments in which ELIXIR RDM services operate. However, it does not seem straightforward to estimate the current number of FTE providing RDM-related services in each Node. The difficulty is mainly due to the lack of a consensus on what RDM services are and which organisation in the Node is providing the service, and the fact that RDM services are often provided as in-kind contributions by professionals in research, such as bioinformaticians, IT experts, software developers or librarians.

**Figure 2.**
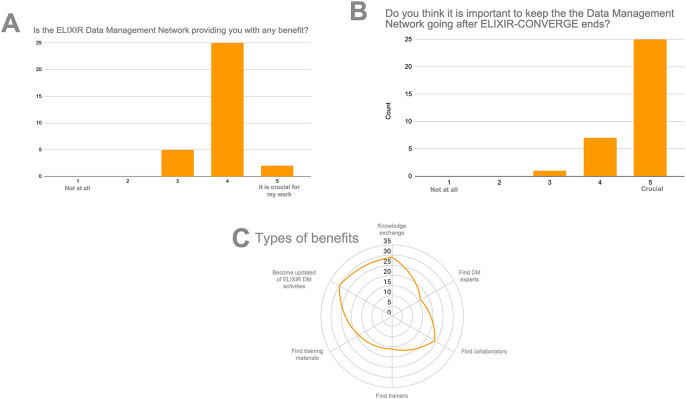
Survey of benefits of a Data Management expert network during the ELIXIR-CONVERGE project. (A) Perceived benefit of the Data Management Network, rated on a scale from 1 (not at all) to 5 (crucial for my work); (B) Importance of keeping the DM Network going after ELIXIR-CONVERGE ends, rated on a scale from 1 (not at all) to 5 (crucial); (C) Numbers of respondents that perceive they obtain different types of benefits by participating in the DM Network.

ELIXIR-CONVERGE brought together RDM professionals from 23 Nodes, creating the Data Management (DM) Network, which currently counted 149 members, of which 37 were coordinators as representatives for each Node (1-2 per Node). The DM Network shared experiences and knowledge about implementing RDM governance, policy, technical infrastructures, business models, etc. in the different Nodes. This information has largely been published in RDMkit as tool assemblies for RDM and on the national resources pages so that it can be used as examples by other Nodes. The DM Network organised focus groups around several RDM topics, where experts discussed and generated guidelines for best practices (e.g. how to find data repositories, how to document data) shared via RDMkit. In a survey designed to evaluate the role of the DM Network among the ELIXIR Nodes involved in ELIXIR-CONVERGE ( Jareborg, 2023), 33 participants from 19 Nodes answered that the Network was clearly beneficial to their work and that keeping the network alive was important to them ( Figure 2). The main benefits perceived by the participants were knowledge exchange, information about RDM initiatives within ELIXIR, and finding experts for collaborations and training.

Similarly, in FAIRplus, almost 100 data professionals from academia (including ELIXIR Nodes), pharmaceutical and information service companies were brought together to share their knowledge on FAIR implementations in the FAIR Cookbook. Collaboration between public and private sectors is essential in and around FAIR RDM matters, and such networks should be cultivated and sustained.

### ELIXIR guidelines and best practices for RDM

The RDMkit, FAIRCookbook and the Data Stewardship Wizard (DSW) are ELIXIR Recommended Interoperability Resources, that are the three main resources of ELIXIR guidance and best practices for RDM.

***RDMkit***

Launched in 2021, RDMkit (the Research Data Management toolkit for life sciences) is a knowledge resource reflecting the evolving best practices and guidelines in RDM. RDMkit was implemented as part of ELIXIR-CONVERGE to help with standardising life science data management across Europe. Professionals in RDM and researchers from ELIXIR Nodes, Communities use RDMkit to provide scoped guidelines that place the relevant life science RDM tools and resources in the context of research. RDMkit guidelines identify RDM issues, provide plausible solutions, recommend relevant tools for RDM activity, and illustrate how these tools have been combined to enable data life cycle management in various domains. RDMkit has integrations with other ELIXIR RDM resources, such as the FAIR Cookbook, the DSW and the ELIXIR registries ( FAIRsharing, bio.tools, TeSS). The knowledge contained in RDMkit has been recognised by the European Commission and recommended as a useful resource in the Horizon Europe Programme Guide and the ERC Guideline. Moreover, ELIXIR Belgium, Norway, and UK have added RDMkit as an ELIXIR Node service (see Node Service Selection Process.)

***FAIR Cookbook***

Launched in 2020, and created by researchers and data management professionals in academia, pharma and information service industries, the FAIR Cookbook covers the key steps in a FAIRification journey, ‘ levels and indicators of FAIRness, a maturity model, the technologies, tools and standards available, as well as the skills required and challenges presented. All with the aim of improving data FAIRness. The recipes are citable, via PIDs, and authors are credited, via their ORCID and the CreDiT ontology. The FAIR Cookbook is cross-referenced with other ELIXIR resources, such as the RDMkit, FAIRsharing, bio.tools, DSW (work in progress) and external resources such as the Pistoia Alliance’s FAIR Toolkit. As described ( Rocca-Serra *et al.*, 2022), the FAIR Cookbook is uniquely positioned not only to serve as practical guidance to improve everyday tasks but is also contributing to a curriculum on FAIR data and informing discussions on the necessary changes to deliver FAIR within organisations. FAIR Cookbook is one of the key outcomes of the FAIRplus project, which connected ELIXIR with industry partners to address hurdles to FAIR implementation by developing practical guidelines, processes, and tools to make data FAIR. The UK, Luxembourg, Swiss and Spain Nodes have now included the FAIR Cookbook in their service delivery plan.

***Data Stewardship Wizard (DSW)***

DSW has been developed by a collaborative effort between ELIXIR Czech Republic and ELIXIR Netherlands, combining knowledge about data stewardship and a cutting-edge questionnaire using dynamic web forms developed by ELIXIR Czech Republic ( Pergl *et al.*, 2019). DSW’s primary goal is to turn data management planning from an obligation into a benefit for a project by easing the process. Several experts in data stewardship have contributed to the latest versions of the question and answer flows in the system. Questions in DSW can use or link to external resources to provide answers. A prime example of this is the suggestion of databases and standards from FAIRsharing, based on responses to certain questions. RDMkit is frequently linked from DSW and the inverse link from RDMkit to the relevant section of DSW is created automatically; links are also being created with recipes in the FAIR Cookbook. DSW is used and recommended by several Nodes and funders. Specifically, ELIXIR Sweden and Norway have adopted DSW, as well as institutions in the Czech Republic, Netherlands, Spain, Denmark, France and Portugal. Funders such as EU Commission (Horizon EU programme guide), ZonMw (Netherlands), UB-BOTT (Norway), TAČR/OP JAK (Czech Republic) also recommend DSW.

### RDM training resources

In 2021, RDM professionals, training experts and trainers from ELIXIR and beyond identified gaps in the Nodes’ RDM training programmes and defined priority topics (DMP, Data Stewardship, FAIR/metadata and Reproducibility) ( Cardona *et al.*, 2021) for training development. Collaborative work from all the ELIXIR Nodes during ELIXIR-CONVERGE resulted in an extensive Data Management/Data Stewardship (DM/DS) course portfolio ( Cardona *et al.*, 2021) ^1^ which aligns with the Nodes’ RDM training strategies. The portfolio includes both generic DM/DS resources and materials, as well as specific materials related to the priority topics mentioned above, and it also includes training materials for specific domains (e.g. the Plant Demonstrator). The course portfolio is available in TeSS, in which training materials are tagged with RDM terms to increase their findability, and links to relevant training materials were included in RDMkit. FAIRsharing has recently launched its FAIRsharing Educational component, in collaboration with EOSC and RDA data champions, across all disciplines. In collaboration with the ELIXIR Training Platform, the learning paths methodology has been applied to RDM for data stewards and researchers (e.g. with the Plants Sciences and the System Biology Communities), and work is ongoing to extend the ELIXIR-GOBLET Train-the-Trainer programme to tackle the challenges that exist in teaching RDM. As a result of these ELIXIR-CONVERGE activities, training expertise and capacity in RDM has been significantly increased in ELIXIR.

### Examples of RDM initiatives in ELIXIR Nodes

Numerous Nodes are involved in or drive national initiatives aimed at capacity building, professionalisation of data stewardship and alignment of RDM practices across universities and institutions. Examples of national RDM initiatives in which ELIXIR Nodes are involved are listed in Table 1.

**Table 1.** Examples of RDM initiatives in ELIXIR Nodes.

ELIXIR Node	Initiative	Description
Belgium	Flemish Research Data Network (FRDN)	• Knowledge Hub: a Research Data Management Community of practice. • Making collaborative content that supports researchers in making their data open and FAIR.
Cyprus (observer)	Cyprus Open Science Initiative	• Recommendation to the National Open Science Cloud initiative in the EOSC governance to include RDM. • Provide technical and policy support for onboarding of service providers into EOSC, including data management.
Finland	National Open Science coordination	• Working group for professionalisation of RDM experts.
France	Recherche Data Gouv	• Ecosystem to share and open research data. • Data management clusters. • Thematic reference centre for Biology and Health.
Germany	National Research Data Infrastructure (NFDI)	• Systematic management of scientific and research data. • Consortia of stakeholders to provide science-driven data services to research communities.
Greece	Hellenic Open Science Initiative	• Recommendations on Open Science practices (including data management) for the national research performing and research funding organisations in Greece.
Italy	Italian Computing and Data Infrastructure	• Open Science Cafe to increase awareness about RDM and Open Science.
Netherlands	Data Stewards Interest Group (DSIG) & Health-RI (health data infrastructure)	• Platform for data professionals (beyond Netherlands) to share experiences. • FAIR data stewardship training and capacity building program for life sciences and health research (incl. ZonMw funded projects).
Norway	BioMedData	• Network of data management experts across research infrastructures. • Capacity building, identification and adoption of best practices.
Portugal	Ready for BioData Management?	• Capacity building program in data management for researchers and institutions. • Offering introductory and advanced courses in DMPs.
Slovenia	ELIXIR-SI RDM expert group initiative	• Capacity building, network of DM/DS experts, best practices. • DM/DMP tools and services.
Sweden	SciLifeLab & Wallenberg National Program for Data-Driven Life Science	• Supporting the RDM needs of the initiative.
Switzerland	Swiss Open Research Data Strategy	• Extending the existing nationwide network of data managers to include data stewards. • Developing a Certificate of Advanced Studies (CAS) in data management and data stewardship.
United Kingdom	ELIXIR-UK DaSH Fellowship FAIRsharing Educational	• RDM capacity building and professionalising data stewardship. • Producing and delivering training in FAIR data stewardship using ELIXIR United Kingdom knowledge. • Working group for professionalisation of RDM experts.

The individuals driving, organising, or delivering these initiatives are part of ELIXIR Nodes. They learn from members of other Nodes about RDM skills, training, and resources, and then contribute to their national initiatives by organising events, managing national communities, providing training, adopting and sharing resources within their institutions, and taking on other leading and practical roles.

## Challenges for RDM professionals in ELIXIR

### Lack of consensus on data steward profiles

Institutions and universities that are part of ELIXIR and beyond recognise the importance of professional data stewards for RDM support for researchers in all stages of the research life cycle. RDM involves a variety of professionals, including research scientists, IT specialists, policy makers, legal staff, libraries, funding agencies and publishers. This leads to a lack of consensus and clarity on the responsibilities, knowledge and skills required for RDM professionals. This confusion stifles effective definition of the profile of a data steward across ELIXIR Nodes. Profiles of data stewards are often developed locally in member organisations of ELIXIR and vary greatly both within and between these organisations. Moreover, some RDM professionals are centralised within Nodes or national centres, whereas others are embedded into member universities and institutes. This poses the question of how these professionals can be brought together into an effective community that recognises and supports these differences.

A formalised ELIXIR RDM Community provides the forum necessary to overcome this lack of consensus and initiate the harmonisation efforts around professionalisation of data stewardship across Nodes. The members of the RDM Community can focus on sharing experiences and practices about how RDM services are implemented in each Node, taking into account the interactions with member Node, universities and institutions. This could be considered as a continuation of the work started by the DM Network in the ELIXIR-CONVERGE project. This exchange of experiences and practices will help to shape career pathways across the different ELIXIR member states and can function as examples for the sector.

The report of the Dutch National Programme Open Science (NPOS) and ELIXIR Netherlands have provided an excellent framework and a comprehensive list of competencies, skills and qualifications defining and classifying the role of the data stewards ( Jetten *et al.*, 2021), as has the Danish National Forum for Research Data Management ( Wildgaard *et al.*, 2020). The RDM Community aims to generalise these, and similar guidelines generated by other Nodes, in order to promote adoption of these profiles within the organisations hosting ELIXIR Nodes and within the respective national environments. The RDM Community will undertake this effort in alignment with current insights of the RDA Professionalising Data Stewardship Interest Group as well as the EOSC Task Force Data stewardship, curricula and career paths.

### Lack of overview of the ELIXIR RDM ecosystem

ELIXIR’s activities impacting the management of research data and software in line with the FAIR (Findable, Accessible, Interoperable Reusable) principles are scattered across projects, Platforms, Communities and Focus Groups. The multitude of ELIXIR’s tools, information resources, registries, databases, training courses and material suitable for the implementation of the FAIR principles in data management (FAIRification journey) are valuable and often recommended by RDM professionals to peers and researchers. However, an overview of how to use the available RDM resources is missing, whether in an integrated fashion (as an ecosystem) or as individual modules. This makes their dissemination, use and application more challenging. Currently, a user of the ELIXIR services (e.g. researchers, RDM professionals, trainers, funders) has to find and explore each resource from its dedicated webpage, and understand when and how each guideline or component relates to another and how they could be used together during the data life cycle, to make data, software or other outputs FAIR. Although this problem has been partially tackled in the ELIXIR Guidelines section, it is by no means a comprehensive representation of how ELIXIR resources could be used as an ecosystem for good RDM and FAIRification throughout a research project.

Therefore, building an ELIXIR RDM Community where its members, who are involved in several ELIXIR initiatives related to RDM, FAIR implementation and assessment, can share their knowledge is essential for the delineation of an overview of the ELIXIR RDM ecosystem. The Community coordinates and advises about RDM initiatives, by highlighting overlaps, identifying synergy and reducing possible duplication of efforts. The RDM Community acts as a single point of reference for RDM knowledge and expertise under the ELIXIR branding.

### Lack of an organisational framework to share and exchange RDM expertise

RDM professionals from different Nodes might have different expertise in specific aspects of research data management (e.g. writing DMPs, managing sensitive data, brokering, assessment and evaluation of FAIRness of digital object etc.), due to differences in the contexts in which Nodes operate. Although this diversity in expertise might be a challenge for the harmonisation effort, it also presents an opportunity for peers to learn from each other and complement their knowledge. However, RDM professionals face the difficulty of finding an effective framework that recognises these differences and supports the sharing of knowledge, particularly in small Nodes that do not receive national funding for such initiatives. Until now, the process of sharing expertise and learning from peers about RDM has only happened during time-limited projects, such as ELIXIR-CONVERGE (e.g. best practices working group, data brokering task group), FAIRplus (e.g. Squads and Fellows) Staff Exchange programme, BioHackathon projects and other RDM related initiatives.

The RDM Community is a structured and long-term framework where RDM professionals can learn new skills and strengthen their expertise by sharing their experience with peers on several practical RDM topics, which affect their day-to-day work. The shared knowledge can then be translated into tangible outputs, such as content for the ELIXIR RDM ecosystem and other materials beneficial for the ELIXIR organisation and the research community at large. These activities help to keep the existing ELIXIR RDM resources relevant and up to date. The RDM Community is a unique opportunity for its members to share expertise, increase their confidence and provide better and more harmonised services for researchers.

### Lack of a sustainable framework for RDM training expertise and activities

During ELIXIR-CONVERGE and FAIRplus, extensive RDM course portfolios have been built, the RDM training expertise in ELIXIR Nodes has increased significantly and a strong RDM trainer network has been formed. All activities have been executed in close collaboration with the ELIXIR Training Platform and benefited from its resources. However, the work is far from complete, and course materials are in continuous need of updating. There is no clear portfolio owner or a proper framework for embedding RDM training activities and the RDM trainer network in the ELIXIR structure. The main RDM training related actions going forward are: (i) gathering the RDM courses in the portfolio into learning paths for specific audiences, thus shaping the ELIXIR RDM curriculum, (ii) increasing findability and reusability of the developed courses and modules by optimising annotation and linking in TeSS, RDMkit and FAIR Cookbook, (iii) encouraging (re) use of the RDM materials in ELIXIR Nodes, and (iv) increasing RDM training capacity in Nodes by finalising and launching the RDM Train the Trainer programme.

As is already the case for many other ELIXIR Communities (Galaxy, Single Cell Omics, Plants etc.), the RDM Community needs to establish a strong formal presence in the ELIXIR Training Platform. Thus, the consolidation and further expansion of RDM training activities in ELIXIR, according to and implementing the Training Platform best practices, will be safeguarded.

## ELIXIR RDM Community

### Members identity

The expected members of the Community are life science RDM professionals in academia and industry in ELIXIR and beyond. It is meant to be inclusive to anyone that has a professional interest in RDM for the life sciences. It shall be open to not only data stewards, but also similar roles like data manager, data architect or data engineer, as well as to interested experts from relevant infrastructure providers (data repositories, computing services and resources etc.) and experts in specific aspects of RDM (IT experts, librarians, policy officers, regulators, software developers, data scientist/analyst, bioinformaticians and trainers). The Community includes discipline or subject specific data stewards. Members should have an interest and an active role in implementing, supporting and promoting good RDM practices to make research data, software and other types of output compliant with Open Science and the FAIR principles.

Examples of activities that the intended Community members engage in are not only direct support to researchers, but also the development and implementation of RDM policies in organisations, as well as the establishment of tools, services, practices and training resources to enable proper data management. Any life science RDM professional that wants to join the Community can do so by registering for membership on the RDM Community web page. Association to ELIXIR is not required for membership.

Members of the ELIXIR RDM Community experience a multitude of benefits that enhance their professional capabilities and collective impact. By engaging with peers across Nodes, members gain valuable insights, share best practices, and exchange knowledge, thereby boosting their confidence and efficiency in delivering harmonised research data management (RDM) services to researchers. This collaborative environment not only strengthens internal cohesion but also serves as a central hub for aligning activities across Nodes, Platforms, and external stakeholders. As a result, members play a pivotal role in harmonising RDM practices, fostering collaboration, and driving innovation within and beyond the ELIXIR network.

### Scope of the Community

Research data management is a broad discipline that, to different extents, affects the activities of most of the existing structures of the ELIXIR organisation. Therefore, it is necessary to outline the role and scope of the ELIXIR RDM Community given the established and ongoing activities in ELIXIR. The overall purpose of the RDM Community is to bring together experts in RDM to develop ELIXIR’s vision, and coordinate its activities, within the domain of research data management according to the FAIR principles. Peers in the Community will share knowledge, experience and practices in order to increase their confidence, efficiency, and deliver more harmonised RDM services for researchers across Nodes.

**Figure 3.**
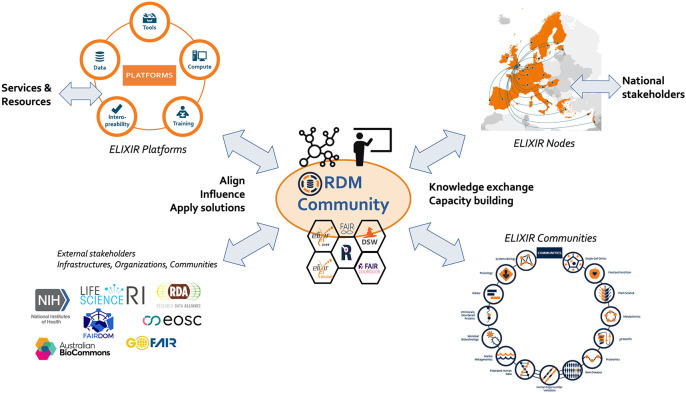
The RDM Community: A focal point for RDM practice in ELIXIR. The RDM Community will engage and coordinate with ELIXIR Nodes, Platforms, Communities, Focus Groups and external stakeholders to promote and drive harmonisation of RDM activities within ELIXIR and beyond.

The responsibilities that are in scope for the Community can be categorised into three main interacting pillars: (i) the network of RDM professionals across all Nodes (and beyond), (ii) the management of RDM knowledge and know-how in the form of the content of the ELIXIR RDM ecosystem, and (iii) the management of the RDM training resources and expertise. As RDM is high on the agenda in the global research community, an important fourth component that goes across the three pillars is the engagement with other external stakeholders that are active in this area. Bringing these four elements under the roof of one ELIXIR RDM Community creates a focal point to harmonise these activities across Nodes, Platforms and Communities, as well as with key external stakeholders outside ELIXIR ( Figure 3).

***The network of RDM professionals***

In the ELIXIR-CONVERGE and FAIRplus projects, networks of life science RDM professionals have been established that include named Node representatives from each ELIXIR Node, as well as other RDM staff in those Nodes. Peers in these networks have shared knowledge and generated a tangible output as guidelines for RDM best practices that mainly reside in RDMkit and FAIR Cookbook. The recognition of RDMkit and the FAIR Cookbook as a valuable source for guidance by the Horizon Europe programme guide and the Innovative Medicines Initiative, as well as the positive feedback given by the DM Network in the ELIXIR-CONVERGE project about its utility, has highlighted the need to establish a home for this group of professionals to continue these activities as a formal structure within ELIXIR, beyond time-bound projects. Providing a long-term framework for RDM professionals across Europe to come together and exchange knowledge is at the heart of an ELIXIR RDM Community. The ELIXIR RDM Community thus supersedes the less formal competence networks established in previous projects. One prioritised aspect of this group is to work on facilitating RDM capacity building in all ELIXIR Nodes, so that the Nodes can provide state-of-the-art RDM services for their local communities while aligning practices across countries and using ELIXIR resources.

***Management of RDM knowledge***

The comprehensive bodies of RDM knowledge that have been gathered by ELIXIR-CONVERGE, FAIRplus and earlier activities are important and valuable resources to guide RDM professionals and researchers in their everyday practice. This pool of knowledge collated by the community for the community, mainly resides in three different knowledge resources with differing scopes and granularity: RDMkit, DSW, and the FAIR Cookbook. Additional guidance on how to use FAIRsharing for RDM has been collected in FAIRsharing Educational. However, the content in these resources is not a static product but needs constant maintenance and expansion over time to remain relevant. It is the responsibility of the RDM Community to provide the maintenance and overall coordination of the content across this RDM ecosystem for FAIR data, as well as disseminating it among Nodes and institutions in the different countries. The activities of the editorial boards of RDMkit and FAIR Cookbook fit well within the remit of the Community, which will bring these resources closer together, for example under a single board, with individual steering groups. The content that the RDM Community shall capture and manage in these resources should comprise those topics that affect the RDM and FAIRness aspects of research outputs, such as data and software.

***Management of RDM training resources and expertise***

In ELIXIR-CONVERGE, an extensive portfolio of training resources has been built, as a basis for the ELIXIR DM/DS Curriculum. However, continuous work is needed to make them (re) usable and keep them up to date. Moreover, during ELIXIR-CONVERGE, a strong RDM trainers network was formed, establishing solid RDM training expertise within the Nodes. This emerging network raised great interest in the Nodes, showing their willingness to work together on developing training materials, sharing their knowledge, and establishing best practices and standards for RDM training.

The consolidation and expansion of this RDM trainers network is in the remit of the RDM Community. Also, there is still a lack of trainers and training, and a collaboration between the RDM Community trainers and the ELIXIR-GOBLET Train-the-Trainer programme is beneficial towards forecasting any pedagogical challenges that will arise when the training is rolled out. There is a clear need for dissemination of RDM know-how, practices and resources and a demand for RDM training material and courses, for researchers, trainers and RDM professionals. This can be solved by the RDM Community in close collaboration with the Nodes and the ELIXIR Training Platform. It is the responsibility of the RDM Community, and the network of RDM trainers within, to coordinate the activities to fulfil these needs. This will enable the upskilling of RDM trainers and scaling up RDM training capacity in the Nodes, while bridging to RDM professionals outside ELIXIR as well.

***External stakeholders***

Apart from the three main pillars above, the responsibility to coordinate the interactions with the increasing number of external international stakeholders in this area is in scope for the RDM Community. This is relevant for the activities of all three main pillars. ELIXIR has an RDA Activities Focus Group with the purpose of enabling ELIXIR members to leverage the benefits of the Research Data Alliance and to ensure that the life sciences point of view is represented in the RDA community. ELIXIR has, through participation in projects, collaborations and Platform activities, engaged with various other stakeholders that are working on solving RDM issues, such as EOSC through the ELIXIR EOSC Focus Group, ESFRIs, international organisations, and national infrastructures outside ELIXIR member states. The FAIRsharing Educational is an example of RDM and FAIR-related information material created under the RDA FAIRsharing WG, and with seed funds from EOSC. It is in the remit of the RDM Community to be responsible for how RDM aspects in these stakeholder activities can be leveraged for the Community, as well as how future developments should be influenced. Engagement with these external global stakeholders both through the various fora for knowledge exchange that they provide, as well as through the possibility for RDM professionals to join and contribute to the Community, will offer the possibility for dissemination and use of the open RDM resources developed and maintained by ELIXIR.

### Out of scope

To set the boundary against other possible activities and ELIXIR services, the following should be considered to be out of scope for an ELIXIR RDM Community: the technical development and provisioning of services in the ELIXIR RDM ecosystem, providing RDM helpdesk services for researchers and consortia, delivering RDM training programmes, and curation of data already deposited in data repositories and knowledge bases. The technical service provisioning of RDMkit, DSW and the FAIR Cookbook shall lie with other parties within ELIXIR. However, the content management contributions for these resources must be done in close collaboration with the technical service providers, to ensure that they are fit for purpose.

The RDM Community will not provide any RDM helpdesk support services. These must reside in the various Nodes and other local organisations, though the activities in the RDM Community will help these organisations to establish such services for their local needs. Likewise, it is not in the remit of the Community to deliver training programmes. The development of training materials and teaching needs considerable time and effort and would be delivered through future (ELIXIR) projects and by the Nodes themselves, leveraging the training resources and expertise of the Community. The Community should, at least initially, focus on promoting FAIR at source approaches to data management and facilitating the researchers’ data journeys. Thus, curation of data already deposited in Deposition Databases and knowledge-based resources would be out of scope.

## Alignment with ELIXIR structures

### ELIXIR Platforms

***Data Platform***

In collaboration with the ELIXIR Data Platform the Community will develop data management best practice guidelines to facilitate the flow of FAIR data from life science research to Core Data Resources (CDR), ELIXIR Deposition Databases (EDD) and ELIXIR Community data resources. It includes establishing guidelines and maturity models for data brokering together with EDDs. Based on the best practice guidelines, the Data Platform and the RDM Community will work together to promote the harmonisation of database formats and standards. The Community will also benefit from the liaison of the Data Platform and the ELIXIR Biocuration Focus Group to exchange knowledge on best practices for data curation and quality assurance in databases and repositories.

***Interoperability Platform***

The ELIXIR Interoperability Platform develops, maintains and promotes interoperability services to help people and machines to discover, access, integrate and analyse biological data. In particular, the platform has also operated in the RDM space, focusing on the services and their use in interoperability stories, and plans on continuing to build the FAIR-enabling portfolio of products, processes and practices. The RDM Community will collaborate with the Platform to incorporate interoperability best practices and guidelines aimed at researchers, RDM professionals and infrastructure providers, into the RDM ecosystem. The Platform has activities aimed at alignment of FAIR service architecture across all ELIXIR Communities as well as the Data Platform and ELIXIR projects. The results of those activities that are related to RDM will be included in the ELIXIR RDM ecosystem, whose content coordination falls under the scope of the RDM Community. While incentivizing interoperability practices in RDM, the Community will promote key services offered by the Interoperability platform such as the ELIXIR Recommended Interoperability Resources (RIRs) (e.g. OLS, FAIRsharing, RightField, ISA, Identifiers.org, etc.), and the Platform initiatives (e.g. Bioschemas, RO-Crate, RightField, etc.). The RDM Community will contribute to the harmonisation of interoperability standards across domains and data resources and work together with the Platform for wider adoption.

***Training Platform***

The RDM trainers network is an integral part of the ELIXIR Training community. As such the RDM Community benefits from and contributes to many ELIXIR Training Platform activities. The RDM Community will continue to expose the information about its training events and materials in TeSS. During ELIXIR-CONVERGE, standardised keywords for RDM training courses and material in TeSS were established to improve the visibility and findability; this work will be further improved. RDM trainers also actively contributed to the work of the FAIR Training Focus Group, both on creating content for courses on FAIR data, as well as on the FAIR Training Handbook. This handbook is currently being developed to train instructors on how to make training materials FAIR, and this should be the basis for the FAIRification of RDM training materials. Furthermore, the RDM trainers will continue using the assessment strategy ( Gurwitz *et al.*, 2020) implemented by the Training Platform, which measures the quality and impact of ELIXIR training events in a coordinated and consistent data collection approach. Finally, RDM Train the Trainer activities will be aligned with the ELIXIR-GOBLET Train-the-Trainer programme. New and expert trainers will continue to build their training skills by following the pedagogical best practices taught in the ELIXIR-GOBLET Train-the-Trainer programme ( Morgan *et al*., 2017) courses. They will also benefit from the materials and expertise developed in ELIXIR-CONVERGE.

***Tools Platform***

The RDM Community will align with the Tools Platform to promote the use of available registries and tools for research data management by describing it in best practices, and establish cross-fertilization actions to exchange best practices for research data management and research software management, e.g. making FAIR software and workflows. The RDM Community will also be in a position to convey user needs and feedback to the Platform and tool providers. The RDM Community and the Tools Platform could work together on guidelines for writing Data and Software Management Plans (DMP/ SMP), particularly for European funders, for projects involving both data and software development. The aim could be to provide researchers with guidelines for an integrated approach to manage both data and software in the same project.

***Compute Platform***

The RDM Community will collaborate with the Compute Platform to write and disseminate best practices to make FAIR software, FAIR data analysis workflows, and secure and trustworthy computing environments. The RDM Community will ensure that computational services about authentication and authorisation, storage and data transfer, and cloud and computing resources are included in the RDM ecosystem and that their use is described from the RDM perspective.

### ELIXIR Communities

The existing ELIXIR Communities are key interaction partners that deal with real-world RDM problems and challenges for their respective domains. They offer a possibility for knowledge exchange and capacity building that goes both ways. The RDM Community can use the challenges of the different Communities to help develop and improve RDM guidelines, solutions, standards, learning paths and training for the different domains, as well as to learn from, and leverage, developments made in the Communities themselves to improve on general RDM principles and solutions so that they stay fit for purpose over time. This in turn will help to drive standards harmonisation across communities, whenever possible.

Several ELIXIR Communities (e.g. Plants, Microbiome, Microbial Biotechnology, Toxicology, etc.) have already used RDMkit, DSW and FAIR Cookbook for dissemination of domain specific standards and RDM best practices. Domain pages in the RDMkit include many of the existing ELIXIR Communities, as well as descriptions of tool assemblies that serve as inspiration for real-world solutions to RDM challenges for many life science domains. One such example is the RDMkit Marine metagenomics domain page by the Microbiome Community that outlines RDM considerations and standards, as well as pointing to the tool assembly page for the Norwegian marine metagenomics tool assembly. The ELIXIR Communities have also engaged with RDM training experts in ELIXIR-CONVERGE to create learning paths targeted to the RDM training needs in the respective scientific domains. One such example is the ELIXIR Plant Sciences Community that has established and enhanced learning paths for the Community with the input of stakeholders outside of ELIXIR and ELIXIR-CONVERGE. The general aspects of this work also feed back to the RDM Community as a framework that can be reused in other domains. We foresee that individuals in the different ELIXIR Communities with an interest in RDM will also be members of the RDM Community, so that good RDM becomes embedded in the activities of the ELIXIR Communities.

### ELIXIR Focus Groups

Two key Focus Groups in ELIXIR with a remit to interact with external stakeholders and that have a prominent focus on research data management are the RDA Activities Focus Group and the EOSC Focus Group. The RDM Community provides a forum for the bidirectional dissemination of the work done in these two Focus Groups. This activity will improve the exchange and uptake of RDM developments made in these external organisations to ensure that ELIXIR aligns with, and makes most use of, state of the art RDM best practice and know-how. This also has the potential to increase the engagement of RDM professionals in these Focus Groups, which will further strengthen the interactions with these important external stakeholders. As part of this, it offers the opportunity to influence even further the RDM developments in the activities of RDA and EOSC. The ELIXIR EOSC Strategy 2022 ( Tedds *et al*., 2022) by the EOSC Focus Group states that “ELIXIR is a partner for EOSC - in implementation and application”. The RDM Community shall strive to take on this aspiration for ELIXIR in the area of research data management, acting as a communication hub for standards, interoperability and data flow related questions between ELIXIR and EOSC. There is also a clear connection to the FAIR Training Focus Group, with which the Community collaborates via the RDM trainers around best practices for FAIRification of digital objects. Several of the other ELIXIR Focus Groups also deal with different RDM aspects. The Community will reach out to these Focus Groups to address research data management questions for their particular domains, e.g. data readiness with the Machine Learning (AI) Focus Group and standards for particular life science domains, such as for the Pathogen Data Focus Group.

## Objectives of the RDM Community

Short-term and longer-term objectives of the RDM Community are listed in Table 2 below. Short-term objectives focus on sustaining and consolidating the outputs of previous initiatives and projects into the unified structure of the RDM Community. Longer-term objectives, instead, aim to tackle the challenges that the Nodes are facing and to work towards a coordinated action of Nodes to ensure a harmonised FAIR Data Management programme for life science.

**Table 2.** Objectives of the RDM Community.

Area	Objective
Short-term (~2 years)	
People network	Provide a forum that enables regular inter-personal knowledge exchange between life science RDM professionals.
	Facilitate ELIXIR Node capacity building in deploying RDM services, e.g. by establishing a knowledge handbook for RDM service providers and developing an RDM services maturity model for ELIXIR Nodes.
RDM knowledge	Coordinate and contribute to the RDM ecosystem content by collaborating with the editorial boards of RDMkit and FAIR Cookbook, to ensure that the content is continuously fit for purpose.
	Collect requirements that would facilitate flow of FAIR data from researchers to repositories (such as the ELIXIR Deposition Databases) via RDM services, in order to improve the FAIRness of research data made available for life science and society at large.
RDM training	Provide a forum that enables knowledge and teaching best practices exchanges among RDM trainers.
	Ensure that RDM training materials are being developed and FAIRified, enabling reuse and adoption by the ELIXIR Nodes.
	Develop RDM learning paths for researchers, data stewards, tool developers and trainers.
External stakeholders	Create and maintain an overview of external stakeholder engagements with RDM aspects across ELIXIR Platforms, Communities, Focus Groups and projects.
Long-term (~3-5 years)	
People network	Implement initiatives to enable a harmonised FAIR data management programme for life science across Nodes.
	Strengthen skills and knowledge of life science RDM professionals in the Nodes.
	Create strong links with national RDM communities to leverage relevant resources or initiatives, and to increase the usage of the ELIXIR RDM ecosystem at the national level.
	Continue to sharpen the professional profile of RDM personnel and enable attractive career pathways within the national and European research sectors.
RDM knowledge	Support ELIXIR structures - Platforms, Communities, and Focus Groups - regarding best practices in their RDM and FAIRification activities.
	Harmonise RDM standards across ELIXIR Communities, wherever possible.
RDM training	Maintain and continuously develop an up-to-date collection of RDM training resources that supports the delivery of the defined RDM learning paths.
	Establish a strong RDM training expertise hub in ELIXIR.
	Ensure all ELIXIR Nodes have sufficient RDM training expertise and capacity through Train the Trainer activities.
External stakeholders	Devise a clear strategy on how to strengthen the relationships to key external stakeholders and how to coordinate RDM aspects across ELIXIR in engagements with these, to influence and align with international developments and solutions in the RDM field.
	Develop and evolve RDM guidance and models with the Life Science RIs and EOSC, acting as a communication hub for standards, interoperability and data flow related questions.
	Engage with national funders to optimise RDM guidance and policy in order to maximise public gain through data deposition and reuse.

## Conclusions

RDM has become the focus point for many funders and institutions that are part of ELIXIR and for the ELIXIR organisation itself. RDM professionals in ELIXIR have demonstrated their RDM expertise by bringing their knowledge, experience, know-how and skills to successful projects that aimed to support RDM. Therefore, the ELIXIR RDM Community, as defined by the scope and the objectives described in this paper, provides the way required to start tackling RDM challenges for the life sciences and to harmonise the approach to FAIR data management across ELIXIR Nodes and beyond.

## Data Availability

No data are associated with this article.

## Footnotes

^1^
Updated numbers (February 2020 - December 2022): 18 Nodes contributed to 119 training events with 2849 participants.
