# Supplementary material for: A research data management (RDM) community for ELIXIR
Source: F1000Res. 2024 Sep 30;13:ELIXIR-230. Originally published 2024 Mar 27. [Version 2] doi: 10.12688/f1000research.146301.2 (PMC11474151; doi:10.12688/f1000research.146301.2)
Supplement: Peer review history

# Reviewer report: Reviewer response for version 2

**DOI:** 10.5256/f1000research.172134.r328117

**Published:** 2024-10-14

**Authors:** Sara El Gebali

**Affiliations:** 1 DataCite, Hannover, Germany

No further comments

Is the topic of the opinion article discussed accurately in the context of the current literature?

Yes

Are arguments sufficiently supported by evidence from the published literature?

Yes

Are all factual statements correct and adequately supported by citations?

Yes

Are the conclusions drawn balanced and justified on the basis of the presented arguments?

Yes

Reviewer Expertise:

Research Data Management, Curation, Semantic web

I confirm that I have read this submission and believe that I have an appropriate level of expertise to confirm that it is of an acceptable scientific standard.

# Reviewer report: Reviewer response for version 2

**DOI:** 10.5256/f1000research.172134.r328116

**Published:** 2024-10-03

**Authors:** Jeff Christiansen

**Affiliations:** 1 Australian BioCommons, Sydney, New South Wales, Australia

Thankyou to the authors for addressing all of my comments on the first version of the manuscript.

 The manuscript now clearly outlines the broad research data management (RDM) landscape across ELIXIR, challenges facing RDM professionals across ELIXIR and beyond; and the objectives and scope of the ELIXIR RDM Community to support life science RDM professionals in Europe and beyond.

Is the topic of the opinion article discussed accurately in the context of the current literature?

Yes

Are arguments sufficiently supported by evidence from the published literature?

Yes

Are all factual statements correct and adequately supported by citations?

Yes

Are the conclusions drawn balanced and justified on the basis of the presented arguments?

Yes

Reviewer Expertise:

Life Science and Bioinformatics Digital Infrastructure to support Research

I confirm that I have read this submission and believe that I have an appropriate level of expertise to confirm that it is of an acceptable scientific standard.

# Reviewer report: Reviewer response for version 1

**DOI:** 10.5256/f1000research.160372.r281726

**Published:** 2024-06-06

**Authors:** Sara El Gebali

**Affiliations:** 1 DataCite, Hannover, Germany

The article “A Research Data Management (RDM) Community for ELIXIR” effectively highlights the pressing need for a coordinated approach to RDM within the ELIXIR infrastructure. By proposing the formation of an ELIXIR RDM Community, the article addresses the current inefficiencies caused by scattered resources and aims to align practices with the FAIR (Findable, Accessible, Interoperable, Reusable) and Open Science principles. The vision for this community is well-articulated and focuses on three main pillars: networking, knowledge management, and training.

 Minor comments:

- While the article lays out a clear vision for the ELIXIR RDM Community, it would benefit from more detailed implementation strategies which include potential challenges and mitigation strategies.
- The interest in making outputs globally available is mentioned but not detailed. The section “Table 2. Objectives of the RDM Community” could include plans for connecting with the wider global community. Efforts in collating available training/RDM material or translating them into other languages like French or Spanish would benefit a wider population, such as those in Africa and Latin America.
- Engagement with External Stakeholders:  This section could be moved closer to the description of the network of RDM professionals. 
- The manuscript lists examples of national RDM initiatives involving ELIXIR Nodes but does not explain the nature of their involvement. Highlighting how ELIXIR contributes to these efforts would strengthen the link between ELIXIR nodes and other national initiatives, showcasing the added value of ELIXIR’s participation 
- Members Benefits: Including a section on member benefits would make the ELIXIR RDM Community more attractive to potential members. This could detail professional development opportunities, access to exclusive resources, and the ability to influence RDM practices on a broader scale.
- To make the community more inviting and keep potential members interested, the manuscript should introduce the expected members earlier on. When readers know right away that they are welcome, they are more likely to stay engaged. Perhaps consider moving this text earlier in the article. 
  -  “The expected members of the Community are life science RDM professionals in academia and industry in ELIXIR and beyond. It is meant to be inclusive to anyone that has a professional interest in RDM for the life sciences. It shall be open to not only data stewards, but also similar roles like data manager, data architect or data engineer, as well as to interested experts from relevant infrastructure providers (data repositories, computing services and resources etc.) “

Figure Legends:

     •    Figure 1: The colors of the points need to be clearly defined to ensure readers understand what each point represents.

     •    Figure 3: The description should be expanded to clearly outline the role of the RDM Community as a focal point for RDM practices within ELIXIR. This will enhance understanding of its central role in coordinating and harmonizing RDM activities.

Is the topic of the opinion article discussed accurately in the context of the current literature?

Yes

Are arguments sufficiently supported by evidence from the published literature?

Yes

Are all factual statements correct and adequately supported by citations?

Yes

Are the conclusions drawn balanced and justified on the basis of the presented arguments?

Yes

Reviewer Expertise:

Research Data Management, Curation, Semantic web

I confirm that I have read this submission and believe that I have an appropriate level of expertise to confirm that it is of an acceptable scientific standard, however I have significant reservations, as outlined above.

# Response

**Published:** 2024-09-18

**Authors:** Munazah Andrabi

**Affiliations:** Computer Science, The University of Manchester, Manchester, England, UK

**We thank the reviewer for the insightful comments. Please, find below our responses to each comment and the references to the revised manuscript.**

**Major comments: **

- **While the article lays out a clear vision for the ELIXIR RDM Community, it would benefit from more detailed implementation strategies which include potential challenges and mitigation strategies.**

**Response**

 We appreciate your suggestion; however, we have decided not to include this information directly in the text. Our focus in this white paper is on the current status quo and the ELIXIR Community roadmap. The implementation will follow ELIXIR and community best practices, primarily through a bottom-up approach, with concrete initiatives aimed at identifying potential challenges and solutions. As we work towards building an active and engaged community, activities will be community-driven, with ongoing assessments of challenges. If necessary, mitigation strategies will be developed as work progresses in each area.

  

- **The interest in making outputs globally available is mentioned but not detailed. The section “Table 2. Objectives of the RDM Community” could include plans for connecting with the wider global community. Efforts in collating available training/RDM material or translating them into other languages like French or Spanish would benefit a wider population, such as those in Africa and Latin America.**

**Response**

 We appreciate your suggestion; however, we have decided not to include this information directly in the text.

 While the interest in making outputs globally available is noted, it is important to clarify our current scope and priorities. Our primary focus at this stage is to establish a strong foundational network within our existing collaborations. We are coordinating the existing RDM community, working closely with other ELIXIR communities and platforms such as the Training Platform, TeSS group, and Interoperability Platforms, which manage and co-develop resources within the RDM ecosystem. We also collaborate with the Focus Groups in Research Data Alliance(RDA), which is our point of contact for global outreach and synergies. Although broader outreach is within our objectives, translating materials into other languages like French or Spanish is not, as such activities can be done better professionally at each Node level. Our current efforts are concentrated on enhancing collaboration and resource sharing within our established network.

- **Engagement with External Stakeholders:  This section could be moved closer to the description of the network of RDM professionals.**

**Response**

 While we appreciate the merit of restructuring the text by moving this section, we have decided to keep the External Stakeholders section in its current place in the texts as we think it fits best as part of the description of the scope of the Community.

  

- **The manuscript lists examples of national RDM initiatives involving ELIXIR Nodes but does not explain the nature of their involvement. Highlighting how ELIXIR contributes to these efforts would strengthen the link between ELIXIR nodes and other national initiatives, showcasing the added value of ELIXIR’s participation**

**Response**

 The type of contribution to national initiatives by Node members has been now highlighted with the addition of the following text under the “Examples of RDM initiatives in ELIXIR Nodes” paragraph:

*The individuals driving, organising, or delivering these initiatives are part of ELIXIR Nodes. They learn from members of other Nodes about RDM skills, training, and resources, and then contribute to their national initiatives by organising events, managing national communities, providing training, adopting and sharing resources within their institutions, and taking on other leading and practical roles.*

- **Members Benefits: Including a section on member benefits would make the ELIXIR RDM Community more attractive to potential members. This could detail professional development opportunities, access to exclusive resources, and the ability to influence RDM practices on a broader scale.**

**Response**

 The paragraph on benefits for members of the RDM Community is now included in the paper. The following text has been added to the “Members identity” paragraph:

*Members of the ELIXIR RDM Community experience a multitude of benefits that enhance their professional capabilities and collective impact. By engaging with peers across Nodes, members gain valuable insights, share best practices, and exchange knowledge, thereby boosting their confidence and efficiency in delivering harmonised research data management (RDM) services to researchers. This collaborative environment not only strengthens internal cohesion but also serves as a central hub for aligning activities across Nodes, Platforms, and external stakeholders. As a result, members play a pivotal role in harmonising RDM practices, fostering collaboration, and driving innovation within and beyond the ELIXIR network.*

- **To make the community more inviting and keep potential members interested, the manuscript should introduce the expected members earlier on. When readers know right away that they are welcome, they are more likely to stay engaged. Perhaps consider moving this text earlier in the article. **

**Response**

 The following text has been added to the Introduction section of the manuscript:

*“The RDM Community is meant to be inclusive to anyone that has a professional interest in RDM for the life sciences in academia and industry both in and outside of ELIXIR.”*

**Comment**

 Figure Legends:

**Figure 1: The colors of the points need to be clearly defined to ensure readers understand what each point represents.**

**Response**

 The colours represent different ELIXIR Nodes. The following sentence has been added to the figure legend to clarify this.

**

*“Colours represent different ELIXIR Nodes* *.”*

**Comment**

**Figure 3: The description should be expanded to clearly outline the role of the RDM Community as a focal point for RDM practices within ELIXIR. This will enhance understanding of its central role in coordinating and harmonising RDM activities.**

**Response**

 The following sentence has been added to the Figure legend:

*The RDM Community will engage and coordinate with ELIXIR Nodes, Platforms, Communities, Focus Groups and external stakeholders to promote and drive harmonisation of RDM activities within ELIXIR and beyond.*

# Reviewer report: Reviewer response for version 1

**DOI:** 10.5256/f1000research.160372.r281723

**Published:** 2024-06-03

**Authors:** Jeff Christiansen

**Affiliations:** 1 Australian BioCommons, Sydney, New South Wales, Australia

ELIXIR is a European network of life science data related infrastructures, that is structured around the concepts of Nodes (collection of research institutes within member countries), Platforms (centred around Data, Tools, Compute, Interoperability and Training infrastructures), and Communities (groups of experts across the ELIXIR Nodes that represent a scientific or technological theme, and drive the development of standards, services, and training in and across ELIXIR).

 In any open science research endeavour, good research data management is central to conducting best practice research, and enabling research data to be made Findable, Accessible, Interoperable and Reusable (FAIR) enables its re-use where possible and appropriate. In recognition of the central importance of Research Data Management (RDM) to research practice, over the past decade there has been considerable investments by ELIXIR and its Nodes into RDM, through various time-boxed projects and initiatives, and these have resulted in the development many tools and resources to assist. There was however (surprisingly) until recently, no dedicated and ongoing channel for RDM professionals across ELIXIR to exchange knowledge and share resources.

 Driven by a need for better coordination, and the generation of sustainable and harmonised RDM practices across ELIXIR, a Research Data Management (RDM) Community within ELIXIR (see https://elixir-europe.org/communities/research-data-management) was recently formed.

 The white paper by D’Anna and co-authors (from 17 ELIXIR Nodes), describes the broad landscape of RDM across ELIXIR (including previous joint ELIXIR efforts and projects); RDM tools and guidelines produced by ELIXIR to date; examples of RDM initiatives across various ELIXIR nodes; challenges facing the many RDM professionals across ELIXIR; the objectives and scope of the ELIXIR RDM Community; and how the RDM Community is intended to align with other ELIXIR components (Platforms, other Communities and Focus Groups).

 Forming the ELIXIR RDM Community represents a very sensible approach to address the ongoing RDM coordination and uplift challenges across the ELIXIR network, as it leverages previous RDM related outputs and many aspects of the existing operational structures of ELIXIR to underpin its operations. It will also essentially to bring together a community of RDM experts to further develop ELIXIR’s vision and coordinate its RDM activities, initially leveraging existing assets, and importantly, to illustrate to researchers and others how to practically use the various ELIXIR RDM resources.

 Overall, the paper is a clear and informative review. It is well written, and logically presented, covering various facets, all of which are relevant for a white paper of this type.

 Major comment:

 The External Stakeholders paragraph briefly describes how the RDM Community and its members may interact with RDM groups beyond ELIXIR through various projects and initiatives. The activities and outputs of this group will however be of great interest outside Europe as improving RDM is a global challenge. Will all outputs/products/guides etc produced by the RDM Community be made available for adoption and adaptation by others outside the ELIXIR network? If so, how will this occur? The manuscript would benefit from some further commentary on the approaches the RDM Community will utilise to enable global shareability/findability of resources they will produce or curate. With respect to this global angle, it would also be useful to understand the membership rules for the ELIXIR RDM Community - i.e. can external stakeholders can join the RDM Community or is membership restricted to those associated ELIXIR Nodes?

 The authors describe the ELIXIR Data Management Network https://elixir-europe.org/about-us/how-funded/eu-projects/converge/wp1/dm-network. How does/will the DM network and RDM Community interact? Will the RDM Community supersede the DM network?

 Minor comments:

 Tense - the ELIXIR RDM Community appears to already exist ( https://elixir-europe.org/communities/research-data-management) but the future tense is often used in the manuscript (e.g. in Abstract (p2), the ‘proposed’ ELIXIR RDM Community ‘will’ bring together; Introduction (p4) the ‘proposed’ ELIXIR RDM Community etc), which is confusing to the reader. If the RDM Community has been established, I would request that the tense of the manuscript is changed to the present tense to reflect this situation.

 Figure 1 Legend - It’s not clear what the colours of the points represent. I assume different ELIXIR Nodes, but can this please be defined in the Figure legend?

 Figure 2 Legend. A self contained legend would be beneficial, to explain that the results are from the 2023 survey of DM Network members.

 P7 “RDMkit has been implemented” > “RDMkit **was** implemented’

 P7 - “ELIXIR Belgium, Norway, Sweden, and UK have added RDMkit as a Node service”. This needs more explanation. What is a “Node service”? This should be defined.

 P7 - “FAIR Cookbook is one of the key outcomes of the FAIRplus project” - link/ref missing for FAIRplus project.

 P7 - It would be useful to note that the RDMkit, FAIRCookbook and DSW are all ELIXIR recommended interoperability resources https://elixir-europe.org/news/resource-announcement-2023

 P7 - “RDM professionals, training experts and trainers from ELIXIR and beyond” > “ **In 2021, **RDM professionals, training experts and trainers from ELIXIR and beyond”

 P9 - “Institutions and universities part of ELIXIR” > “Institutions and universities **that are** part of ELIXIR”

 P10. “Therefore, building an ELIXIR RDM Community where its members, which are involved” > “Therefore, building an ELIXIR RDM Community where its members, **who** are involved”

 P10. “particularly in small Nodes that do not receive national fundings for such initiatives” > “particularly in small Nodes that do not receive national **funding** for such initiatives”.

Is the topic of the opinion article discussed accurately in the context of the current literature?

Yes

Are arguments sufficiently supported by evidence from the published literature?

Yes

Are all factual statements correct and adequately supported by citations?

Yes

Are the conclusions drawn balanced and justified on the basis of the presented arguments?

Yes

Reviewer Expertise:

Life Science and Bioinformatics Digital Infrastructure to support Research

I confirm that I have read this submission and believe that I have an appropriate level of expertise to confirm that it is of an acceptable scientific standard, however I have significant reservations, as outlined above.

# Response

**Published:** 2024-09-18

**Authors:** Munazah Andrabi

**Affiliations:** Computer Science, The University of Manchester, Manchester, England, UK

We thank the reviewer for the insightful comments. Please, find below our responses to each comments and the references to the revised manuscript. 

**Major comment:**

 The External Stakeholders paragraph briefly describes how the RDM Community and its members may interact with RDM groups beyond ELIXIR through various projects and initiatives. The activities and outputs of this group will however be of great interest outside Europe as improving RDM is a global challenge. Will all outputs/products/guides etc produced by the RDM Community be made available for adoption and adaptation by others outside the ELIXIR network? If so, how will this occur? The manuscript would benefit from some further commentary on the approaches the RDM Community will utilise to enable global shareability/findability of resources they will produce or curate. With respect to this global angle, it would also be useful to understand the membership rules for the ELIXIR RDM Community - i.e. can external stakeholders can join the RDM Community or is membership restricted to those associated ELIXIR Nodes?

***Response***

***Dissemination of outputs to a global audience:***

*All outcomes from the RDM Community will be open and accessible to all and published in open resource repositories like Zenodo. We will also be actively disseminating content and expertise  via international conferences like the ECCB. In addition, various ELIXIR nodes are part of international projects like BY-COVID, BioIndustry 4.0 and EOSC Focus groups. Outside the EU ELIXIR actively engages with the NIH in USA and Australian BioCommons. Our resources like RDMkit and FAIRCookbook are already in use by these institutions.*

***The following paragraph has been added to the paper, in the “scope of the community - external stakeholders” section:***

*“Engagement with these external global stakeholders both through the various fora for knowledge exchange that they provide, as well as through the possibility for RDM professionals to join and contribute to the Community, will offer the possibility for dissemination and use of the open RDM resources developed and maintained by ELIXIR.”*

**Membership rules:**

 Anyone interested can register  via the link (community page).

 The following sentence with the relevant link has been added to the manuscript, under the “members identity” paragraph:

*“Any life science RDM professional that wants to join the Community can do so by registering for membership on the* *RDM Community web page* *. Association to ELIXIR is not required for membership.”*

**Comment**

 The authors describe the ELIXIR Data Management Network https://elixir-europe.org/about-us/how-funded/eu-projects/converge/wp1/dm-network. How does/will the DM network and RDM Community interact? Will the RDM Community supersede the DM network?

**Response**

 The DM Network was created and active during ELIXIR-CONVERGE as a time-bound project activity. The RDM Community is a continuation of, and supersedes, the DM Network as a formal structure ELIXIR.

 The following sentences have been added in the “Scope of the community - The network of RDM professionals” paragraph to clarify that the DM Network was a network during the project, and that the RDM Community supersedes the DM Network.

*“The ELIXIR RDM Community thus supersedes the less formal competence networks established in previous projects.”*

**Minor comments:**

 Tense - the ELIXIR RDM Community appears to already exist ( https://elixir-europe.org/communities/research-data-management) but the future tense is often used in the manuscript (e.g. in Abstract (p2), the ‘proposed’ ELIXIR RDM Community ‘will’ bring together; Introduction (p4) the ‘proposed’ ELIXIR RDM Community etc), which is confusing to the reader. If the RDM Community has been established, I would request that the tense of the manuscript is changed to the present tense to reflect this situation.

**Response**

 The tense of the text wherever applicable has been changed to reflect the existence of the RDM Community instead of referring to future establishment.

**Comment**

 Figure 1 Legend - It’s not clear what the colours of the points represent. I assume different ELIXIR Nodes, but can this please be defined in the Figure legend?

**Response**

 Yes, colours represent different ELIXIR Nodes. The following sentence has been added to the figure legend to clarify this.

*“Colours represent different ELIXIR Nodes* *.”*

**Comment**

 Figure 2 Legend. A self contained legend would be beneficial, to explain that the results are from the 2023 survey of DM Network members.

**Response**

 The following clarifying sentence has been added to the beginning of the figure legend:

*“Survey of benefits of a Data Management expert network during the ELIXIR-CONVERGE project.”*

**Comment**

 P7 “RDMkit has been implemented” > “RDMkit **was** implemented’

**Response**

 Changed as suggested.

**Comment**

 P7 - “ELIXIR Belgium, Norway, Sweden, and UK have added RDMkit as a Node service”. This needs more explanation. What is a “Node service”? This should be defined.

 The following link to the official definition of Node Service has been included in the manuscript

Node Service Selection Process.

 We noticed that Sweden was incorrectly listed as one of the Nodes that included RDMkit as Node service, but it was an oversight. We have now corrected the text in the manuscript by deleting Sweden from the list.

**Comment**

 P7 - “FAIR Cookbook is one of the key outcomes of the FAIRplus project” - link/ref missing for FAIRplus project.

**Response**

 Hyperlinks are provided the first time a resource is mentioned in the document. To maintain readability and avoid redundancy, we do not include hyperlinks each time the resource is referenced again later in the text. FAIRplus and FAIR Cookbook are hyperlinked already in the Introduction.

**Comment**

 P7 - It would be useful to note that the RDMkit, FAIRCookbook and DSW are all ELIXIR recommended interoperability resources https://elixir-europe.org/news/resource-announcement-2023

**Response**

 It has been added to the “ELIXIR guidelines and best practices for the RDM” section.

**Comment**

 P7 - “RDM professionals, training experts and trainers from ELIXIR and beyond” > “ **In 2021, **RDM professionals, training experts and trainers from ELIXIR and beyond”

**Response**

 Changed as suggested

**Comment**

 P9 - “Institutions and universities part of ELIXIR” > “Institutions and universities **that are** part of ELIXIR”

**Response**

 Changed as suggested.

**Comment**

 P10. “Therefore, building an ELIXIR RDM Community where its members, which are involved” > “Therefore, building an ELIXIR RDM Community where its members, **who** are involved”

**Response**

 Changed as suggested.

**Comment**

 P10. “particularly in small Nodes that do not receive national fundings for such initiatives” > “particularly in small Nodes that do not receive national funding for such initiatives”.

**Response**

 Changed as suggested.
